# The Protective Effect of the Polysaccharide Precursor, D-Isofloridoside, from *Laurencia undulata* on Alcohol-Induced Hepatotoxicity in HepG2 Cells

**DOI:** 10.3390/molecules25051024

**Published:** 2020-02-25

**Authors:** Shengtao Yang, Mei-Fang Chen, Bomi Ryu, Jiali Chen, Zhenbang Xiao, Pengzhi Hong, Shengli Sun, Di Wang, Zhong-Ji Qian, Chunxia Zhou

**Affiliations:** 1 College of Food Science and Technology, School of Chemistry and Environment, Guangdong Ocean University, Zhanjiang 524088, China; 15766385620@163.com (S.Y.); meifangchen93@163.com (M.-F.C.); jiali1745@163.com (J.C.); xzhenbang@163.com (Z.X.); hongpengzhigdou@163.com (P.H.); xinglsun@126.com (S.S.); wangdi@gdou.edu.cn (D.W.); 2 Southern Marine Science and Engineering Guangdong Laboratory, Zhanjiang 524025, China; 3 Department of Marine Life Sciences, Jeju National University, Jeju 63243, Korea; ryu.bomi@gmail.com; 4 Shenzhen Institute of Guangdong Ocean University, Shenzhen 518114, China

**Keywords:** D-Isofloridoside, HepG2 cells, ROS, oxidative stress, apoptosis

## Abstract

Alcoholic liver disease (ALD) threatens human health, so it is imperative that we find ways to prevent or treat it. In recent years, the study of polysaccharides has shown that they have different kinds of bioactivities. Among them are many biological effects that have been attributed to polysaccharide precursors. D-Isofloridoside (DIF) is one of the polysaccharide precursors from the marine red alga *Laurencia undulata*. This study evaluated the effect of DIF on alcohol-induced oxidative stress in human hepatoma cells (HepG2). As a result, DIF attenuated alcohol-induced cytotoxicity, reduced the amount of intracellular reactive oxygen species (ROS), and effectively reduced alcohol-induced DNA damage in HepG2 cells. In addition, a western blot showed that, after DIF treatment, the expression levels of glutathione (GSH), superoxide dismutase (SOD), and B-cell lymphoma-2 (bcl-2) increased, while the expression levels of γ-glutamyl transferase (GGT), BCL2-associated X (bax), cleaved caspase-3, and mitogen-activated protein kinase (p38 and c-Jun N-terminal kinase) signal transduction proteins reduced. This showed that DIF may protect cells by reducing the amount of intracellular ROS and inhibiting intracellular oxidative stress and apoptotic processes. Finally, molecular docking demonstrated that DIF can bind to SOD, GGT, B-cell lymphoma-2, and bax proteins. These results indicated that DIF can protect HepG2 cells from alcohol-induced oxidative stress damage, making it an effective potential ingredient in functional foods.

## 1. Introduction

Alcoholic beverages have played a role in human diet since ancient times. Alcohol is known to negatively affect more than 60 health conditions [1]. According to the World Health Organization (WHO) data from 2014, the harmful use of alcohol results in nearly 3.3 million deaths per year, accounting for 5.1% of the global deaths due to disease. In 2018, alcoholic liver disease (ALD) was ranked by the WHO as the second most common cause of death in humans each year. Long-term excessive drinking is the main cause of ALD [2]. ALD symptoms, including alcoholic fatty liver and alcoholic hepatitis, can further cause steatohepatitis, liver fibrosis, cirrhosis, and the most severe liver cancer [3]. Liver fat accumulation occurs in the early stage of ALD, and only this stage can be reversed by temperance without any medical intervention. Prior to the occurrence of irreversible liver damage, early diagnosis and correct treatment of ALD are essential for the successful treatment of ALD [4].

Long-term excessive drinking can accumulate reactive oxygen species (ROS) produced by alcohol metabolism, which cause oxidative stress in the liver [5]. As highly active molecules, ROS not only play an important role in cell function, but are also closely related to pathological effects. High levels of ROS can disrupt the structure of cells by causing oxidation of nucleic acids, proteins, and lipids, leading to cell death [6]. Research suggests that oxidative stress plays a key role in ethanol-induced liver damage and ALD [7]. Alcohol metabolism is always accompanied by the production of ROS in the liver, which triggers lipid peroxidation, glutathione (GSH) depletion, and malnutrition [8]. Therefore, the inhibition of ROS content and increase of GSH activity may be an effective way for the treatment of ALD. Among them, ROS can be scavenged by superoxide dismutase (SOD), which is an important antioxidant metalloenzyme in vivo [9]. GSH, as an important antioxidant, can scavenge free radicals in vivo [10,11]. SOD and GSH play important roles in alcohol-induced liver oxidative stress. In addition, γ-glutamyl transferase (GGT) is considered to be a sign of excessive drinking in the diagnosis of ALD [12]. Moreover, ROS can serve as a second messenger in the intracellular signaling cascade, and regulate the expression of apoptotic genes through MAPK activation, thereby increasing apoptosis. Caspase-3, bcl-2, and BCL2-associated X protein (bax) have been reported to play a key role in apoptosis [13].

More and more natural drug active molecules have been discovered and studied in depth, especially molecules isolated from marine organisms. As a producer in the marine environment, seaweed was widely studied. As research on seaweed continues to increase, a large number of bioactive molecules (terpenes [14], alkaloids [15], proteins [16], polysaccharides [17], phenolic compounds [18], halogenated compounds [19], etc.) have been isolated from different types of seaweed. In recent years, these molecules have produced fruitful research in terms of their biological properties. Although polysaccharides have been found to have many good biological activities, high-purity algal polysaccharides cannot be obtained due to the complex structure, large number of branches, and different connection methods of seaweed polysaccharides. Research into the activity of seaweed polysaccharides is limited. Some scholars believe that the biological effects of these compounds are attributable to polysaccharide precursors [20], including D-isofloridoside (DIF). 

Floridoside, an early product of carbon fixation, is a carbon source for the synthesis of sulfated cell wall polysaccharides [21]. Sulfated polysaccharides and cell-bound polysaccharides together make up 70% of the algae biomass in *Porphyridium* [22]. Li [21] found that the complete floridoside molecule was directly incorporated by the cell without chemical degradation by the ^14^C pulse tracking technology. Floridoside is probably broken down first into intact free sugars in the cell wall dissolution process [23,24]. Thus, we can get DIF by extraction, separation, and purification. Floridoside and D-isofloridoside are both extracted from the red alga *Laurencia undulata* [25], and are also isomers with significant antioxidant activity. At the same time, there are subtle differences due to the different attachment positions. It is worth noting that D-isofloridoside can direct scavenging of cellular ROS [25].

Evidence has shown that non-poisonous compounds extracted from natural marine foods and herbs have the effect of preventing ALD [26,27,28]. As a bioactive compound, floridoside has received increasing attention. Floridoside has antioxidative [25], anti-inflammatory [29], bone growth-stimulating [30], and neuroprotective activities [31], but its anti-apoptotic activity and protective effects on alcohol-induced liver injury have not been extensively reported. In order to prove that D-isofloridoside (DIF) can be used as a potential preventive substance for ALD, this experiment used alcohol induction to measure the role of DIF in alcohol-induced oxidative stress by measuring the relative cell viability and ROS content. Western blotting was used to measure oxidation and apoptosis-related proteins, and a comet assay was used to determine DNA damage. Finally, molecular docking was used to confirm the mechanism of action of DIF and proteins. A graphical abstract briefly illustrates the synthetic pathway of DIF in the organism, as well as the experimental research ideas and processes in this study.

## 2. Results

### 2.1. Cell Viability of HepG2 Cells

**Figure 1:**
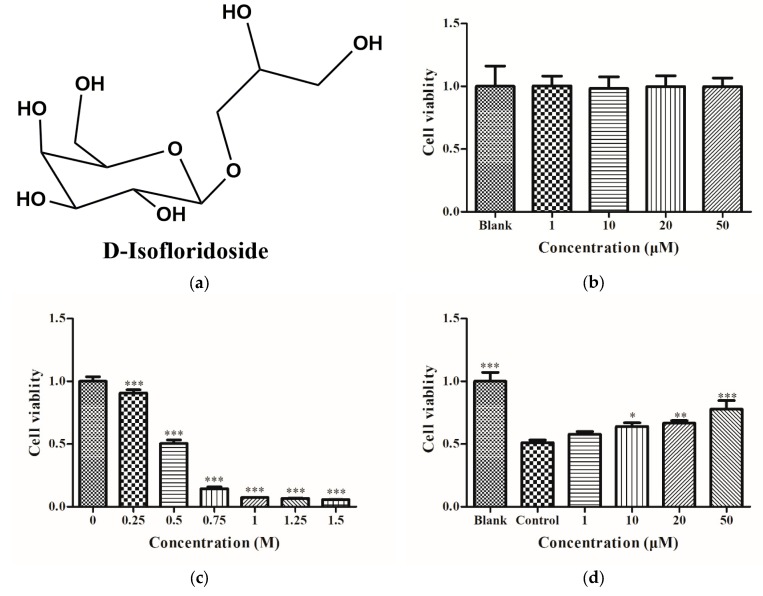
(**a**) Chemical structure of D-isofloridoside (DIF) from *Laurencia undulata*. (**b**) Cytotoxic effect of DIF on HepG2 cells. (**c**) Cytotoxic effect of ethanol on HepG2 cells. (**d**) Protective effect of DIF on HepG2 cells. The blank group was not treated with alcohol and the control group was treated with alcohol. Both groups were not treated with DIF. Data are shown as mean ± SD (n = 3). * Compared with the control group, *p* < 0.05. ** Compared with the control group, *p* < 0.01. *** Compared with the control group, *p* < 0.001.

After treating HepG2 cells with DIF at 1, 10, 20, and 50 μM for 24 h, the relative viability of the cells was determined by the 3-(4,5-Dimethylthiazol-2-yl)-2,5-diphenyltetrazolium bromide (MTT) assay. The results of MTT analysis showed no significant change in cell viability (Figure 1b), indicating that DIF had no significant cytotoxic effect on HepG2 cells. After treating cells with ethanol at different concentrations (0, 0.25, 0.5, 0.75, 1, 1.5, 1.75, and 2 M) for 24 h, the relative viability of the cells was determined by MTT. As depicted in Figure 1c, the relative cell viability decreased in a dose-dependent manner. When the relative cell viability was about 50%, the concentration of ethanol was 0.5 M. HepG2 cells were treated with DIF for 2 h and then treated with 0.5 M ethanol for 24 h. The relative cell viability was determined by MTT. Figure 1d shows that the relative viability of HepG2 cells in the control group after alcohol treatment was significantly reduced compared to the control group; the relative viability of HepG2 cells after DIF treatment was increased compared with the control group. These results indicated that DIF had no toxic effect on cells at a concentration of 0‒50 µM and can reduce ethanol-induced HepG2 cell damage.

### 2.2. Determination of Intracellular ROS

**Figure 2:**
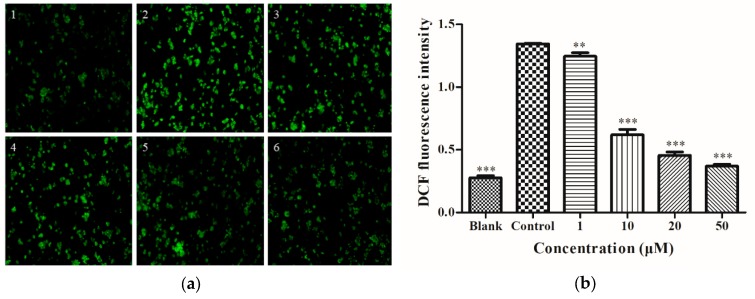
(**a**) Effect of DIF on intracellular reactive oxygen species (ROS) level. (1) HepG2 cells without treatment (the blank group); (2) cells exposed to 0.5 M ethanol (the control group); (3)–(6) cells pretreated with DIF (1, 10, 20, and 50 µM) for 2 h and then treated with 0.5 M ethanol for 24 h. (**b**) The relative DCF fluorescence intensity. Data are shown as mean ± SD (n = 3). ** Compared with the control group, *p* < 0.01. *** Compared with the control group, *p* < 0.001.

The cells were treated as shown in Figure 1d, then treated with 2′,7′-Dichlorodihydrofluorescein diacetate (DCFH-DA) for 20 min, and imaged using an inverted fluorescence microscope to obtain Figure 2a. In the blank group (Figure 2a1), there was no significant fluorescence. On the other hand, in the control group (Figure 2a2), high ROS levels were observed. Treatment with different concentrations of DIF (Figure 2b) for 2 h downregulated ROS levels in a dose-dependent manner. This showed that, at the cellular level, DIF can prevent HepG2 cells from alcohol-induced oxidative injury.

### 2.3. SOD, GSH, and GGT Protein Levels

**Figure 3:**
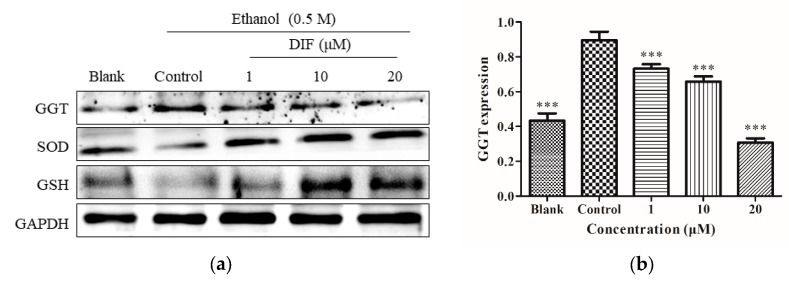

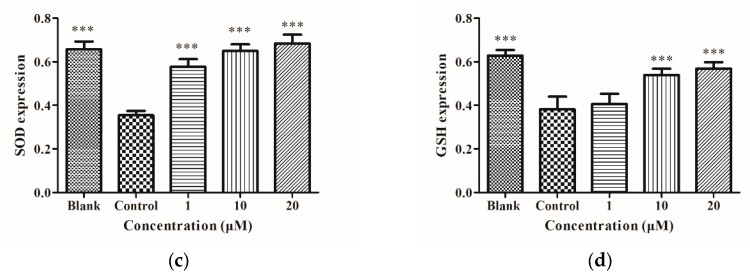
(**a**) Effect of DIF on superoxide dismutase (SOD), glutathione (GSH), and γ-glutamyl transferase (GGT) proteins levels in ethanol-induced HepG2 cells. Cells were treated with DIF (1, 10, and 20 μM) for 2 h, and then treated with 0.5 M ethanol for 24 h. GAPDH was used as an internal control. Protein expression (relative to GAPDH) was evaluated. (**b**) GGT protein expression was evaluated. (**c**) SOD protein expression was evaluated. (**d**) GSH protein expression was evaluated. Data are shown as mean ± SD (n = 3). *** Compared with the control group, *p* < 0.001.

HepG2 cells were treated as shown in Figure 1d, proteins were collected, and then Figure 3a was obtained by Western blotting. As shown in Figure 3, compared to the blank group, the protein levels of the GGT control group increased significantly, while the protein levels of the SOD and GSH control groups significantly decreased; compared with the control group, when DIF was 1, 10, and 20 µM, GGT protein level was downregulated, while SOD and GSH levels were significantly upregulated in HepG2 cells. The above results showed that, at the protein level, DIF can lower ethanol-induced oxidative stress by regulating the production of antioxidant enzymes in HepG2 cells.

### 2.4. Determination of Intracellular DNA Damage

**Figure 4:**
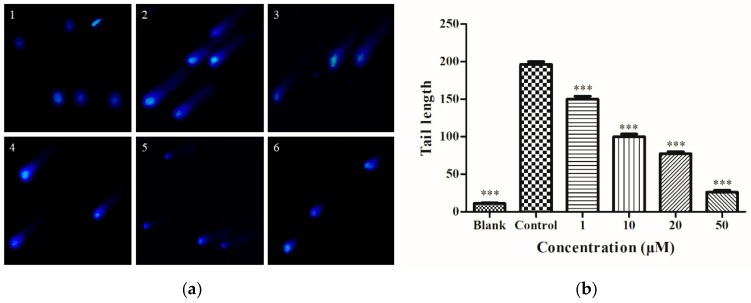
(**a**) Determination of intracellular DNA damage. (1) HepG2 cells without treatment (the blank group); (2) cells exposed to 0.5 M ethanol (the control group); (3)–(6) cells treated with DIF (1, 10, 20, and 50 µM). (**b**) The tail length was analyzed. Data are shown as mean ± SD (n = 3). *** Compared with the control group, *p* < 0.001.

HepG2 cells were treated as shown in Figure 1d, stained with DAPI by comet assay, and then imaged using an inverted fluorescence microscope to obtain Figure 4a. In the blank group (Figure 4a1), there was no significant tailing fluorescence. In the control group (Figure 4a2), HepG2 cells showed significant tailing fluorescence with 0.5 M ethanol. In Figure 4b, as the DIF concentration increased, the length of the comet tail decreased. These results demonstrated that, at the cellular level, DIF has a preventive effect on DNA damage and is dose-dependent.

### 2.5. Caspase-3, bcl-2, and Bax Protein Levels

**Figure 5:**
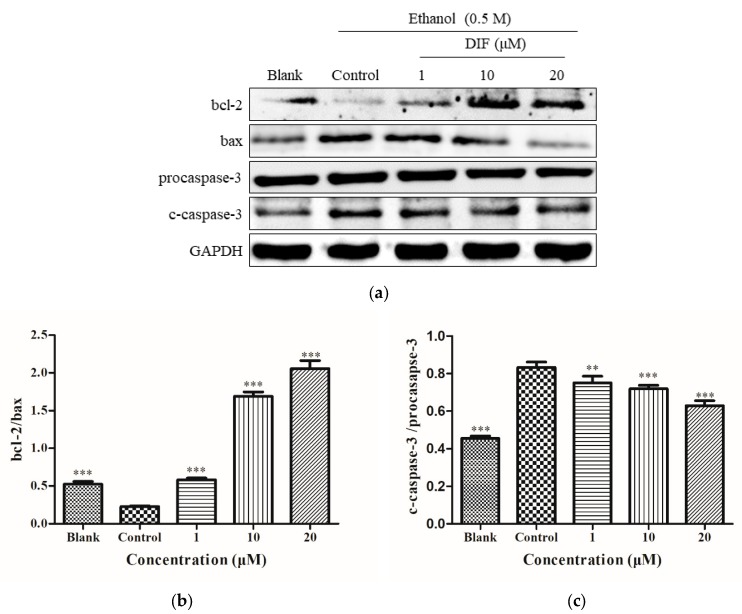
(**a**) The expressions of apoptotic proteins caspase-3, bcl-2, and bax. Cells were treated with DIF (1, 10, and 20 μM) for 2 h, and then treated with 0.5 M ethanol for 24 h. (**b**) The ratios of bcl-2 and BCL2-associated X protein (bax) were calculated. (**c**) The ratios of cleaved c-caspase-3 and procaspase-3 were calculated. Data are shown as mean ± SD (n = 3). ** Compared with the control group, *p* < 0.01. *** Compared with the control group, *p* < 0.001.

The HepG2 cell proteins were processed as shown in Figure 3a, and Figure 5a was obtained. In Figure 5a, compared with the blank group, the expression of bax and cleaved c-caspase-3 proteins was increased, and the expression of bcl-2 was reduced in the control group. After DIF treatment, the expression of bax and c-caspase-3 decreased, while that of bcl-2 increased. In Figure 5b, the value of bcl-2/bax increased significantly. In Figure 5c, the value of c-caspase-3/procaspase-3 decreased. The results for the apoptotic proteins bcl-2, bax, and c-caspase-3 indicated that, at the protein level, DIF can reduce alcohol-induced oxidative stress by inhibiting apoptosis.

### 2.6. JNK and p38 Protein Levels

**Figure 6:**
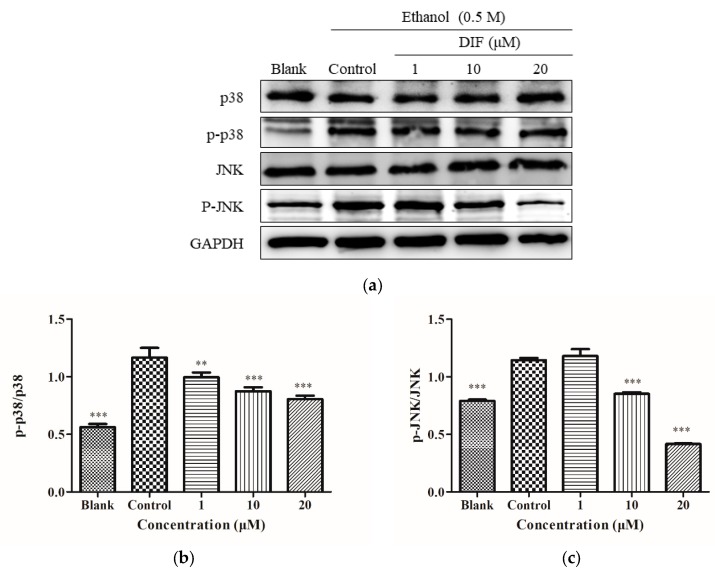
(**a**) The phosphorylation levels of p38, p-p38, JNK, and p-JNK proteins in HepG2 cells. Cells were treated with DIF (1, 10, and 20 μM) for 2 h, and then treated with 0.5 M ethanol for 24 h. (**b**) The ratios of p-p38/p38 were calculated. (**c**) The ratios of p-JNK/JNK were calculated. Data are shown as mean ± SD (n = 3). ** Compared with the control group, *p* < 0.01. *** Compared with the control group, *p* < 0.001.

The HepG2 cell proteins were processed as shown in Figure 3a, and Figure 6a was obtained. The effect of DIF on p38 and JNK was investigated. As shown in Figure 6a, compared with the blank group, the phosphorylation levels of p38 and JNK in the control group were increased significantly. In parts b and c of Figure 6, with DIF treatment, the value of p-p38/p38 and p-JNK/JNK showed a dose-dependent reduction. This showed that, at the protein level, DIF inhibited the phosphorylation level of p38 and JNK.

### 2.7. Bcl-2, Bax, SOD, and GGT Molecular Docking Analysis

The Discovery Studio (Omaha, NE, USA) software was used to simulate the binding sites of DIF and bcl-2, bax, SOD, and GGT proteins, and the CDOCKER interaction energy value was obtained. Furthermore, DIF mainly forms hydrogen bonds between proteins. The proteins docked in this paper were bcl-2, bax, SOD, and GGT. 

**Figure 7:**
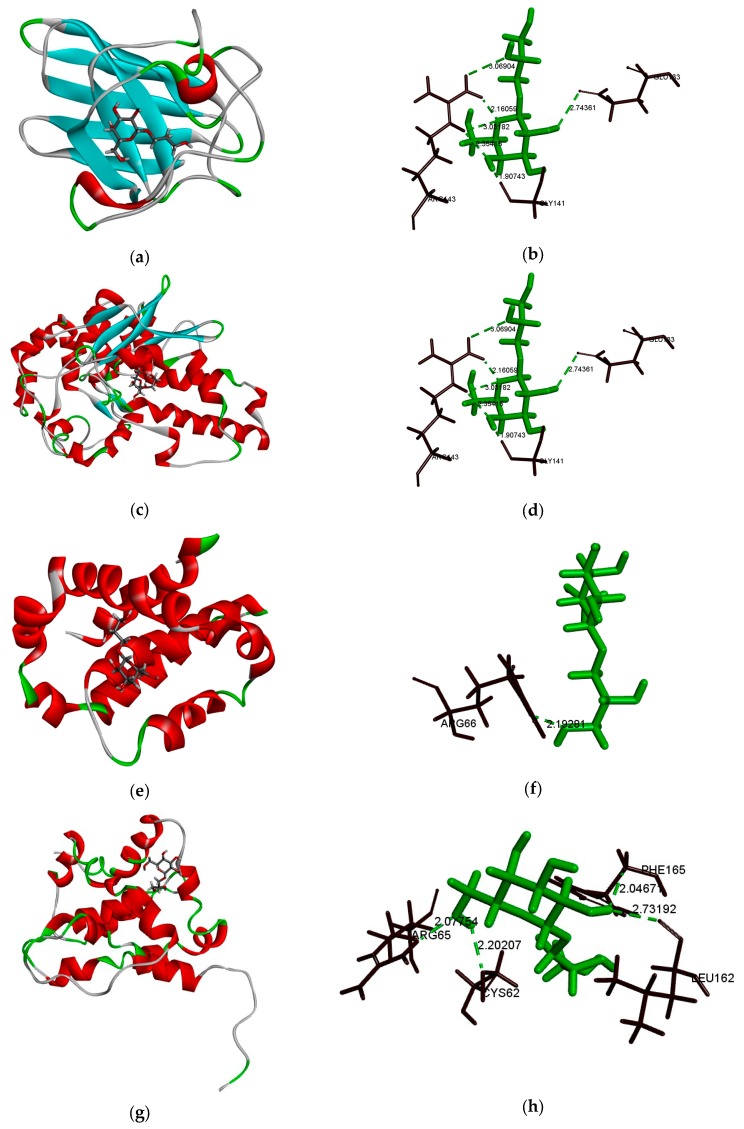
(**a**,**c**,**e**,**g**) 3D model of the interaction between DIF and SOD, GGT, bcl-2, and bax, respectively. (**b**,**d**,**f**,**h**) 3D model of the optimal docking structure interaction between DIF and the active site of SOD, GGT, bcl-2, and bax, respectively.

As shown in Table 1 and Table 2, the number 1 of each model was the maximum value of the docker interaction energy. According to the pairing rules of molecular docking, the maximum value of docker interaction energy is the optimal docking structure. The optimal binding structure of DIF with bcl-2, bax, SOD, and GGT proteins was obtained. 3D models a, c, e, and g in Figure 7 show the optimal docking structures of SOD, GGT, bcl-2, and bax, respectively.

In the interaction between DIF and SOD (Figure 7b), there were five hydrogen bonds (Glu133, Gly141, and Arg143) and the docking score was 29.0355 kcal/mol. In the interaction between DIF and GGT (Figure 7d), there were three hydrogen bonds (Asn344, Glu372, and Thr375) and the docking score was 34.7056 kcal/mol. In addition, DIF combined the amino acid residue Arg66 of bcl-2 protein (Figure 7f) to form a hydrogen bond and its docking score was 29.5175 kcal/mol. DIF combined amino acid residues Cys62, Arg65, Leu162, and Phe165 of the bax protein (Figure 7h) to form four hydrogen bonds and its docking score was 25.6085 kcal/mol. The results of molecular docking showed that DIF may have binding sites with SOD, GGT, bcl-2, and bax proteins. It also suggested that DIF may regulate oxidative stress and apoptosis in vivo by affecting the activities of SOD, GGT, bcl-2, and bax proteins.

## 3. Discussion

In 1954, the structure of floridoside, which is a α-D-galactopyranosylglycerol, was first put forward by Putman and Hassid [32]. This heteroside is involved in osmoregulation [21,33]. Its intracellular content is proportional to the external osmotic pressure. Floridoside, as one of the main products of carbon fixation during photosynthesis [34], is a precursor of cell wall polysaccharides [35].

Li’s research showed that the carbon metabolic pathway first synthesizes floridoside, then produces low-molecular-weight light absorption products, and finally synthesizes sulfated cell wall polysaccharides [21]. Thus, it has been suggested that floridoside can serve as a terminal carbon sink to polysaccharides vying for carbon, or as a short-term carbon reservoir to the newly fixed carbon of polysaccharides, mucus synthesis, protein synthesis, and other cellular compounds [36,37,38]. Previous studies have shown that floridoside can promote osteogenic differentiation and suppress pro-inflammatory responses [29,30]. D-Isofloridoside has the activity of scavenging free radicals, inhibiting ROS expression, and inhibiting MMP-2 and MMP-9. It has not been reported in terms of alcohol-induced oxidative stress.

This article studied the effect of DIF on alcohol-induced oxidative stress in HepG2 cell damage. The graphical abstract briefly illustrates the technical route of the experiment. HepG2 cells were selected as the cell model, and the non-cytotoxic concentration of DIF and the IC_50_ value of ethanol to HepG2 were determined by MTT testing. Secondly, the protective effect of DIF on oxidative stress was evaluated by measuring the expression of ROS, GGT, SOD, and GSH, and the degree of DNA damage. In addition, the expression of apoptotic proteins and MAPK protein was detected, revealing the pathway by which DIF inhibits intracellular oxidative stress. Finally, the possible binding sites of DIF and related proteins were explored through molecular docking.

DIF, at a concentration of 0‒50 μM, showed no cytotoxic effect. The IC_50_ value of ethanol was 0.5 M. The relative cell activity was significantly increased by the addition of DIF (1–50 µM), which proved that DIF had a significant reparative effect on alcohol damage. The ROS detection results showed that the ROS expression of HepG2 cells increased after ethanol treatment; however, treatment with DIF can reduce the production of ROS and GGT proteins, increase the levels of SOD and GSH proteins, and reduce DNA damage. Research has proved that alcohol can lead to oxidative stress and the overexpression of ROS [11,39,40]. Overexpression of ROS induces apoptosis by activating caspase family proteins and other signaling molecules.

As a member of the cysteine protease family, caspase plays a key role in the process of apoptosis [41]. Caspase-3 is considered to be a key protein in the most distal effector pathway of apoptosis [42] and plays a key role in the process of apoptosis. It is well known that apoptosis can be controlled by various apoptosis-related proteins, including bcl-2 family proteins, death receptors, and caspase [43]. The value of cleaved-caspase-3/procaspase-3 and bcl-2/bax was detected by western blots in this study. Results showed that DIF reduced the expression of bax and increased the expression of bcl-2 to reduce alcohol-induced apoptosis.

In the process of ROS overexpression, ROS can serve as a second messenger in the intracellular signaling cascade, which regulates the expression of apoptotic genes through MAPK activation, leading to apoptosis [44]. Mitogen-activated protein kinases, mainly ERK1/2, JNK, and p38, play key roles in regulating cell death, apoptosis, proliferation, and inflammation [45,46,47]. ERKs play a central role in cell proliferation and differentiation, while JNK and p38 are involved in stress response and apoptosis [48]. The results of this study indicated that the phosphorylation levels of p38 and JNK proteins were upregulated during alcohol-induced apoptosis, indicating that alcohol activated the MAPK pathway. Studies have shown that the MAPKs pathway can be activated by alcohol and cause apoptosis in HepG2 cells [49]. DIF can significantly reduce the phosphorylation of JNK and P38, thereby reducing alcohol-induced apoptosis.

Excessive alcohol intake can lead to overexpression of ROS, leading to oxidative stress and causing apoptosis [50,51]. SOD and GGT play an important role in oxidative stress, while bcl-2 and bax have a very important role in cell apoptosis. Therefore, this experiment investigated the binding affinity of DIF to SOD, GGT, bcl-2, and bax though molecular docking. The molecular docking experiment demonstrated the possibility of DIF binding to bcl-2, bax, GGT, and SOD at the molecular level, and further explained the potential of DIF to prevent alcohol-induced liver damage. In this experiment, there were a few possible binding sites for DIF and bax proteins. This may be because bax proteins can be docked with polysaccharides and release active small molecules on polysaccharides. DIF does not have a complete docking structure. As a precursor of polysaccharides, DIF can be used as the terminal carbon of the polysaccharides and can be one of the released active molecules. This process is one of the directions for further research.

A review of the existing research results on DIF, combined with the experimental results of this study, shows that DIF has a protective effect on alcoholic-induced liver injury and is a potential active molecule for the prevention of ALD.

## 4. Materials and Methods

### 4.1. Materials

In a previous study, we purified D-isofloridoside (DIF, Figure 1a) from *Laurencia undulata* [25]. D-Isofloridoside: ^1^H NMR (400 MHz, DMSO-*d*_6_) δ_H_ 4.86 (1H, d, *J* = 2.8 Hz, H-1), 4.58 (1H, m, HO-6), 4.57 (1H, m, HO-4), 4.47 (1H, m, HO-3′), 4.37 (1H, m, HO-3), 4.36 (1H, m, HO-2), 4.45 (1H, m, HO-1′), 3.78 (1H, t, *J* = 2.6 Hz, H-5), 3.71 (1H, brs, H-2), 3.63 (2H, m, H-3′), 3.74 (2H, m, H-3, H-4), 3.66 (2H, m, H-6), 3.49 (3H, m, H-1′, H-2′); ^13^C NMR (100 MHz, DMSO-*d*_6_) δ_C_ 101.3 (C-1), 72.4 (C-2′), 71.8 (C-1′), 71.1 (C-5), 70.3 (C-3), 70.1 (C-4), 69.0 (C-2), 62.5 (C-6), 62.3 (C-3′); LREIMS *m/z* 255.20 (0.09) [M + H]^+^, 163.10 (13.42) [M‒C_3_H_3_O_3_]^+^, 91.05 (36.74) [M‒C_6_H_11_O_4_]^+^. The purity of DIF was greater than 98%, based on the peak area of the component absorbed at each specific wavelength in HPLC analysis [52,53,54]. All cell culture chemicals and the bicinchoninic acid (BCA) assay kit were bought from Thermo Fisher Scientific, Inc. (Waltham, MA, USA). 3-(4,5-Dimethylthiazol-2-yl)-2,5-diphenyltetrazolium bromide (MTT), dimethyl sulfoxide (DMSO), and 2,7-dichlorodihydrofluorescein diacetate (DCFH-DA) were provided by Sigma-Aldrich (St. Louis, MO, USA). The monoclonal antibodies and secondary antibodies were provided by Santa Cruz Biotechnology Inc. (Santa Cruz, CA, USA). All other chemicals and solvents were of analytical grade.

### 4.2. Cell Culture

HepG2 cells were used as an experimental model because they have many characteristics of normal human hepatocytes. HepG2 cells were purchased from the Cell Bank of the Chinese Academy of Sciences (Shanghai, China). HepG2 cells were cultured in Dulbecco’s modified Eagle’s medium (DMEM), fetal bovine serum (FBS), 100 µg/mL of streptomycin, and 100 units/mL of penicillin in a humidified incubator of 5% CO_2_ at 37 °C.

### 4.3. Cell Viability Assay

HepG2 cells were cultured in 96-well plates (4 × 10^5^ cells/mL, 100 µL) for 24 h. This was followed by fresh serum-free medium and DIF (1–50 µM) for 24 h. Medium was removed and 200 µL of MTT (1 mg/mL) was added to each well. The plate was then incubated for 4 h at 37 °C. Subsequently, MTT was removed and 200 µL of DMSO was added to dissolve the formazan crystals. The absorbance was measured using a microplate reader (BioTek, Winooski, VT, USA) at 570 nm. Cell viability was calculated by the formula: cell viability = (OD_DIF_ − OD_Blank_)/(OD_Control_ – OD_Blank_) × 100%. The data were then analyzed by GraphPad Prism5 (GraphPad Prism Software Inc., La Jolla, California, USA). 

### 4.4. Cell ROS Analysis

DCFH-DA has the ability to cross the cell membrane. In the cytoplasm, this dye can be hydrolyzed by intracellular esterase and rapidly converted into a fluorescent dye (DCF) by ROS. The ROS content is directly proportional to the fluorescence intensity [55].

Cells were cultured in 24-well plates, and DIF (1‒50 µM) was added for 2 h. Cells were treated with 0.5 M ethanol for 24 h in a CO_2_ incubator. Subsequently, DCFH-DA (10 µM, 200 µL) was added for 20 min at 37 °C in the dark. Finally, the fluorescence intensity was examined under an inverted fluorescence microscope (Olympus, Tokyo, Japan). Image J (Version 1.46r, NIH, Bethesda, MD, USA) was used to detect the fluorescence intensity in the image and to export the data. Finally, the data were plotted using GraphPad Prism5 software.

### 4.5. Western Blot

The treated cells were collected, 100 μL of RIPA lysis buffer containing 1% PMSF was added, and the cells were subsequently lysed on ice for half an hour. The supernatant was collected at 4 °C for further analysis. The BCA protein assay kit was used to quantify the sample. An equal amount of protein (20 or 40 μg) was used for electrophoresis. The target protein was transferred to a nitrocellulose (NC) membrane (Boston, Mass, USA) by using SDS-PAGE. The membrane was visualized by blocking with 5% skim milk for 2 h, incubating the primary antibodies overnight, and performing secondary antibody incubation with an enhanced chemiluminescence (ECL) detection system (Syngene, Cambridge, UK). Image J (version 1.46r, NIH, Bethesda, Maryland, USA) was used to detect the band brightness in images and to export the data. The data were normalized with internal parameters and plotted using the GraphPad Prism5 software. 

### 4.6. Comet Assay

The comet assay procedure was performed as described by Lu [56]. In brief, cells were treated as described above. The bottom gel was prepared using a 0.8% normal-melting-point agarose (NMA) solution. The cells were then treated with EDTA-trypsin to form a cell suspension (200 cells/µL). The cell suspension and 1% low-melting-point agarose (LMA) were mixed and dropped onto the bottom gel. After the gel was cured, the slides were immersed in a pre-cooled lysate (LS: 2.5 M NaCl, 100 mM Na_2_EDTA, 10 mM Tris, 200 mM NaOH, pH 10, 1% sodium lauryl sarcosinate, and 1% Triton X-100) at 4 °C for 90 min. The slides were then gently immersed in an alkaline electrophoresis solution (AES: 200 mM NaOH, 1 mM Na_2_EDTA, pH > 13) to initiate DNA unwinding. Next, electrophoresis was performed and the slides were stained with DAPI in the dark for 5 min. Finally, the fluorescence intensity was observed under an inverted fluorescence microscope. The comet assay software project (CASP Version 1.2.3 beta1, CaspLab.com, Wratislavia, Lower Silesia, Poland) was used to detect the tail length in the comet analysis and export the data. Finally, the graph was produced using the GraphPad Prism5 software.

### 4.7. Molecular Docking

First, we constructed the 3D structure of DIF, and then found the 3D structure of the desired receptor through the Protein Data Bank (PDB). The 3D models of bcl-2, bax, SOD, and GGT were obtained from the PDB (ID: 4IEH, 2LR1, 1PU0, and 4GG2, respectively). Then, molecular docking was performed using the CDOCKER module feature of the Discovery Studio 2016 software. The CDOCKER module is mainly based on the CHARMm docking program, which uses soft nuclear potential and an optional grid representation to interface ligand molecules to the receptor active sites. CHARMm is a widely recognized and applied molecular dynamics simulation program for the simulation of biological macromolecules, including energy minimization, molecular dynamics, and Monte Carlo simulation [57].

The molecular model of DIF was introduced, and the molecular structure was optimized by hydrogenation, CHARMm force field, and Momany‒Rone charge. Meanwhile, the introduced receptor molecules were structurally optimized, and the active center was identified and defined. Secondly, the CDOCKER module was used to randomly search the conformation of DIF molecules by the high-temperature kinetics method, and then the simulated active site region in each conformation was optimized by simulated annealing to make the docking result more accurate.

Through the above molecular docking study, the structural model of docking and the value of the CDOCKER interaction energy corresponding to the structural model can be obtained. The greater the value of the CDOCKER interaction energy, the lower the energy required for binding and the higher the probability of binding. Therefore, the docking structure obtained, when the CDOCKER interaction energy value is the largest, is the optimal docking structure.

### 4.8. Statistical Analysis

Image J (Version 1.46r, NIH, Bethesda, MD, USA), GraphPad Prism5 (GraphPad Prism Software Inc., La Jolla, CA, USA), Discovery Studio (Omaha, NE, USA), and the comet assay software project (CASP Version 1.2.3 beta1, CaspLab.com, Wratislavia, Lower Silesia, Poland) were used for data analyses. All data were analyzed by one-way ANOVA accompanied by Dunnett’s multiple comparison test for group comparison. Data are expressed as the mean ± SD (n = 3).

## 5. Conclusions

In summary, the current research results show that DIF can increase the expression of SOD and GSH, downregulate the levels of GGT and ROS, and reduce DNA damage. Therefore, ethanol-induced oxidative stress can be prevented by DIF treatment in HepG2 cells. DIF can prevent ethanol-induced apoptosis by upregulating the expression of bcl-2, downregulating the expression of bax and caspase-3, and inhibiting the activation of the JNK and p38 MAPK pathways.

The above experimental results showed that DIF can protect the liver by preventing alcohol-induced oxidative stress and apoptosis of hepatocytes. Therefore, DIF has the potential to be used as a functional hepatoprotective food and a preventive substance for ALD. Although this study demonstrated that DIF has antioxidant and preventive effects on alcoholic liver injury, further research is needed on the mechanism of its expression to determine the signal transduction pathway whereby DIF prevents alcoholic liver injury.

## Figures and Tables

**Table 1:** CDOCKER interaction energy values of the binding sites of DIF to SOD and GGT proteins.

Number	Ligand	Receptor	-CDOCKER Interaction Energy (kcal/mol)
1	DIF	SOD	29.0355
2			28.4436
3			28.3838
4			27.5988
5			26.8758
6			26.1050
1		GGT	34.7056
2			32.5408
3			31.8090
4			29.3496
5			26.5751
6			26.0123
7			24.6752

**Table 2:** CDOCKER interaction energy values of the binding sites of DIF to bcl-2 and bax proteins.

Number	Ligand	Receptor	-CDOCKER Interaction Energy (kcal/mol)
1	DIF	bcl-2	29.5175
2			29.0723
3			28.5170
4			28.3632
5			28.1850
6			27.7746
7			27.6238
1		bax	25.6085
